# Higher Hepcidin Levels in Adolescents with Obesity Are Associated with Metabolic Syndrome Dyslipidemia and Visceral Fat

**DOI:** 10.3390/antiox10050751

**Published:** 2021-05-09

**Authors:** Reyna Rodríguez-Mortera, Russell Caccavello, Ricardo Hermo, María Eugenia Garay-Sevilla, Alejandro Gugliucci

**Affiliations:** 1 Department of Medical Science, University of Guanajuato, Leon 37320, Mexico; ln.reynarm@gmail.com (R.R.-M.); marugaray_@hotmail.com (M.E.G.-S.); 2 Glycation, Oxidation and Disease Laboratory, Department of Research, College of Osteopathic Medicine, Touro University California, Vallejo, CA 94592, USA; russell.caccavello@tu.edu (R.C.); rhermo1055@gmail.com (R.H.)

**Keywords:** hepcidin, ferritin, dyslipidemia, obesity, metabolic syndrome, atherosclerosis

## Abstract

Tightly regulated iron metabolism prevents oxidative stress. Hepcidin is a hormone that regulates iron flow in plasma; its production is induced by an iron overload and by inflammation. It inhibits iron entry into the circulation by blocking dietary absorption in the duodenum, the release of recycled iron from macrophages and the exit of stored iron from hepatocytes. Varied signals responding to iron stores, erythropoietic activity and host defense converge to regulate hepcidin production and thereby affect iron homeostasis. Although it is known that hepcidin increases when interleukin 6 (IL-6) increases, the relationship between hepcidin, dyslipidemia, insulin resistance (IR) and visceral adiposity index (VAI) in adolescents with obesity is unclear. In this cross-sectional study of 29 obese adolescents and 30 control subjects, we explored the difference of hepcidin, iron metabolism markers and IL-6 between obese and non-obese adolescents, and identified associations with inflammation, atherogenic dyslipidemia and IR. As compared to lean controls, obese participants showed 67% higher hepcidin: 14,070.8 ± 7213.5 vs. 8419.1 ± 4826.1 pg/mL^c^; 70% higher ferritin: 94.4 ± 82.4 vs. 55.1 ± 39.6 pg/mL^a^ and 120% higher IL-6: 2.0 (1.1–4.9) vs. 0.9 (0.5–1.3) pg/mL^d^. Transferrin, soluble transferrin receptor and total body iron (as measured by sTFR/ferritin, log10 sTFR/ferritin ratio and sTFR/log ferritin ratios) were not different between the two cohorts. In the whole cohort, hepcidin correlated with VAI (*r* = 0.29^a^), sd-LDL (*r* = 0.31^b^), HOMA-IR (*r* = 0.29^a^) and IL-6 (*r* = 0.35^c^). In obese adolescents hepcidin correlated with TG (*r* = 0.47^b^), VLDL-C (*r* = 0.43^b^) and smaller LDL2 (*r* = 0.39^a^). Hepcidin elevation in adolescents with obesity is linked more to inflammation and metabolic alterations than to iron metabolism since the other markers of iron metabolism were not different between groups, except for ferritin. Studies addressing the long-term effects of higher hepcidin levels and their impact on subclinical anemia and iron status are warranted. ^a^ *p* < 0.05; ^b^ *p* < 0.01, ^c^ *p* < 0.001 ^d^ *p* < 0.0001.

## 1. Introduction

Hepcidin is a hormone that regulates iron flow in plasma; its production is induced by iron overload and by inflammation [1,2]. It inhibits iron entry into the circulation by blocking dietary absorption in the duodenum, the release of recycled iron from macrophages and the exit of stored iron from hepatocytes [3]. Varied signals responding to iron stores, erythropoietic activity and host defense converge to regulate hepcidin production, and thereby affect iron homeostasis [3]. Hepatocytes are the predominant producers of hepcidin. More hepcidin is produced by hepatocytes when iron is abundant, limiting further iron absorption and release from stores [4].

Perhaps for its evolutionary advantage, IL-6 acts as a hepcidin stimulator, depriving some bacteria of iron. However, due to the epidemic of chronic inflammation, the effect of IL-6 has gone awry and may be detrimental.

Although hepcidin is expressed mainly in the liver, studies indicate that it can also be expressed in macrophages and adipose tissue under the conditions of inflammation [5,6].

It is precisely when metabolic syndrome (MetS), which is a complex metabolic disorder, is present that the role of hepcidin as a regulator of iron overload may be subverted and become detrimental to iron balance. One of the main factors implicated in these processes is ectopic fat: that is, visceral, intramyocellular, epicardic or hepatic in non-alcoholic fatty liver disease (NAFLD) [6]. Visceral fat plays a critical role, as the inflammation produced by adipocyte hypertrophy and consequent hypoxia and death leads not only to the secretion of cytokines by the adipocytes themselves, but also in larger amounts from the macrophages that surround them [6]. Compared with subcutaneous fat, visceral adipose tissue is more densely infiltrated by macrophages (crown structures) secreting inflammatory cytokines which include IL-6. Moreover, visceral fat secretes IL-6 into the portal circulation, which flows directly to the liver. In all likelihood, elevated IL-6 in portal blood stimulates hepatic hepcidin synthesis when central obesity is present [7,8,9]. In this case, a signal that should be physiologically protective to deprive bacteria from iron may become detrimental and affect iron metabolism. Indeed, it has been shown that in women between 18 and 55 years of age higher visceral fat is associated with higher hepcidin levels and a tendency to iron deficiency, whereas this does not occur with subcutaneous fat [6].

Some studies conducted in children and adolescents report an iron deficiency secondary to obesity associated with elevated levels of hepcidin [7,8,9]. Obesity in adolescents can be accompanied by dyslipidemia, insulin resistance (IR) and chronic low-grade inflammation, with increased levels of proinflammatory cytokines such as IL-6. This inflammation has been related to IR, type 2 diabetes and cardiovascular risk; however, the mechanisms are not fully elucidated.

We have recently shown that even in the absence of metabolic syndrome (MetS), there is IR-associated dyslipidemia with increased levels of triglycerides (TGs), very-low-density lipoproteins (VLDLs) and small dense low-density lipoprotein (sd-LDL), as well as early endothelial function alterations such as impaired flow-mediated dilation (FMD) in obese vs. lean adolescents [10]. Although it is known that hepcidin rises when IL-6 increases, the relationship between hepcidin, dyslipidemia, IR and visceral adiposity in adolescents with obesity is unclear.

To fill this gap, in this follow-up study we set out to explore the difference of hepcidin, iron metabolism markers and IL-6 between obese and not obese adolescents, and to identify associations with inflammation, atherogenic dyslipidemia, IR and visceral fat. Anthropometric indices, which indirectly indicate body fat and obesity, predict MetS but are poorly sensitive to detect visceral fat. Waist circumference (WC) is used to diagnose abdominal obesity, but it is strongly correlated with body mass index (BMI). Another recently introduced index for the assessment of visceral fat is the visceral adiposity index (VAI). It is based on two anthropometric indices (WC and BMI) and two biochemical markers, triglycerides and high-density lipoprotein cholesterol (HDL-C), and corrects for gender [11]. We therefore employed the VAI in our study.

## 2. Materials and Methods

### 2.1. Participants

This study employed samples from a parent cross-sectional clinical study with 29 obese and 30 lean adolescents from León, Guanajuato, México. Participants were male and female adolescents between 15 and 19 years old with obesity and lean adolescents with Tanner Stage IV and V. The sample size was calculated with a power of 0.80 and an alpha of 0.05.

The study was approved by the Ethics Committee of the University of Guanajuato CIBIUG-P-28-2015; both adolescents and their parents or tutors signed an informed consent form.

### 2.2. Anthropometric Measures

Anthropometric measurements and vital signs were measured as shown previously [10]. Adolescents with obesity were considered to have a BMI greater than the equivalent of 30 kg/m^2^ for an adult. The visceral adiposity index (VAI) was calculated according to Amato et al. [11].

### 2.3. Biochemical Analyses

A venous blood sample was obtained after twelve hours of fasting. Serum was processed the same day and used for the measurement of glucose and lipids using enzymatic methods as shown earlier [10,12]. All other biomarkers were measured by ELISA as follows: Human Hepcidin Quantikine ELISA Cat# DHP250 and Human IL-6 Quantikine ELISA Cat# D6050 were from R&D Systems, NE, Minneapolis, MN, USA. Human Ferritin SimpleStep ELISA Cat# ab200018, Human Transferrin ELISA Cat# ab108911 and Human Transferrin Receptor SimpleStep ELISA Cat# ab217780 were from Abcam, Cambridge, MA, USA.

### 2.4. Statistical Analysis

Normal distributions were tested by the Shapiro–Wilk test. Results are expressed as mean ± SD for continuous variables with normal distribution and as the median and interquartile range for variables with a skewed distribution. The difference between groups was tested by the Student’s *t*-test or Mann–Whitney U. Pearson’s correlation or Spearman’s rank correlation coefficient analyses were used to determine univariate correlation. All analyses were performed using Statistica 7 software (Statsoft Inc., Tulsa, OK, USA). Significance was defined as a *p*-value < 0.05.

## 3. Results

### 3.1. Anthropometric, Clinical and Metabolic Findings

Table 1 shows the anthropometric, clinical and metabolic findings of our participants.

By design, weight, body mass index (BMI), waist and hip circumference ratio, (WC/WH ratio), visceral adipose index (VAI) and fat (%) were significantly different in obese vs. lean adolescents as previously reported. This is an ancillary study with the same population reported in a previous article [10], where other data (lipids, lipoproteins, soluble receptor for advanced glycation products etc.) can be found.

### 3.2. Adolescents with Obesity Display Insulin Resistance

Fasting glycemia was not significantly different between groups: 4.94 ± 0.54 vs. 5.04 ± 0.59 mmol/L, lean vs. obese NS. HbA1c % tended to be higher: 4.74 ± 0.66 vs. 5.05 ± 0.57, *p* 0.054.

Insulin and HOMA-IR were 2-fold higher in obese adolescents. Insulin was 7.78 (6.48–10.14) vs. 15.64 microIU/mL, lean vs. obese, *p* < 0.001. HOMA-IR was 1.78 (1.31–2.20) vs. 3.30 (2.54–5.33), *p* < 0.001. Insulin values and HOMA-IR underscore the presence of compensation of the insulin resistance: adolescents with obesity need 2-fold higher insulin levels to achieve fasting euglycemia.

### 3.3. Adolescents with Obesity Display Higher Levels of Hepcidin and IL-6 without Apparent Changes in Total Body Iron (TBI)

Table 2 shows that adolescents with obesity have significantly higher levels of hepcidin and IL-6 (67% and 100% respectively), as well as higher levels of ferritin (70%).

Higher hepcidin levels may correspond to inflammation or to iron overload. Higher ferritin may reflect iron overload or inflammation. To rule out the putative role of iron status, we also measured transferrin and soluble transferrin receptor in order to discard subclinical anemia due to higher hepcidin on the one hand, and iron overload on the other. Transferrin levels and soluble transferrin receptor were not different between the groups, discarding subclinical anemia. We employed the ratios that correlate with total body iron (TBI) using current hematological recommendations that take into account the measurement of soluble transferrin receptor (sTFR) as a very sensitive index when paired with ferritin: sTFR/ferritin, log10 sTFR/ferritin and sTFR/log ferritin [13]. These are far more sensitive than standard iron and transferrin saturation classic methods, and display a very good correlation with the invasive gold standard for iron stores that requires bone marrow aspiration with iron staining, which is obviously out of the question for a study like this.

All three ratios were not significantly different between obese and lean adolescents, which argues for an apparent dissociation between the higher hepcidin levels we found and iron status—that is, if the higher hepcidin levels were due only to iron status, we would have found evidence of iron overload that would justify it.

### 3.4. Hepcidin Levels in Adolescents Are Associated with Visceral Adiposity, IR and Cardiometabolic Dyslipidemia

Hepcidin levels in adolescents correlated with IL-6 levels, *r* = 0.41, *p* < 0.01.

**Figure 1:**
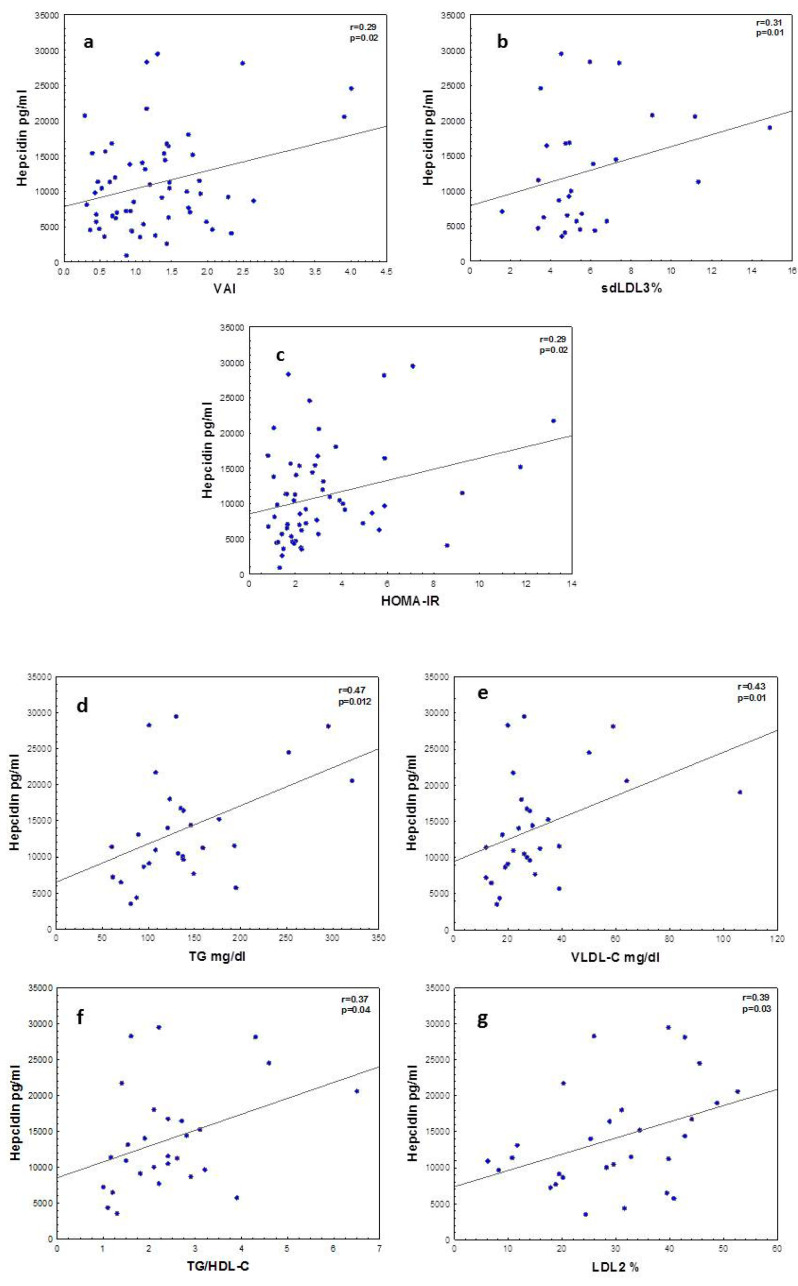
Correlations between hepcidin, insulin resistance and lipid metabolism. (**a**) Hepcidin vs. visceral adipose index (VAI), (**b**) hepcidin vs. small LDL (sd-LDL), (**c**) hepcidin vs. HOMA-IR, (**d**) hepcidin vs. TG, (**e**) hepcidin vs. VLDL-C, (**f**) hepcidin vs. TG/HDL-C and (**g**) hepcidin vs. smaller LDL2. Correlations among variables with normal distributions are shown as Pearson’s correlation coefficients and for variables with skewed distribution Spearman’s correlation coefficients are used.

Figure 1 shows significant correlations between hepcidin and VAI in the whole cohort. There was an association between insulin resistance as measured by HOMA-IR and hepcidin.

Hepcidin correlated with lipid metabolic parameters in obese adolescents. The associations between hepcidin and TG, TG/HDL ratio, VLDL-C and LDL2 (smaller sized LDL) may indicate a link between atherogenic dyslipoproteinemia and hepcidin levels.

## 4. Discussion

This cross-sectional study of adolescents with obesity without overt MetS showed higher hepcidin and IL-6 levels than those of their lean counterparts. Hepcidin correlated with VAI, atherogenic dyslipidemia and IR. The main novelty of the study is that it provides the first evidence in adolescents for a connection between hepcidin levels and MetS dyslipidemia in a framework of increased VAI and IL-6.

Our previous study confirmed an alteration in the classic lipid profile in obese adolescents, evidencing significantly higher levels of TG and TG/HDL-C as well as significantly lower levels of HDL-C, as shown in the prevalence of smaller LDL particles [12]. This is compatible with the classic profile of MetS in adults. In the present study we set out to expand these findings by exploring the status of IL-6 as a potent adipokine from visceral fat that induces hepatic IR and dyslipidemia, since it is known that it may affect iron metabolism as reported by others [4,7,8]. While several studies have shown higher hepcidin levels in adolescents with obesity and the resulting impact on iron status [14,15], none addressed the connection with lipoprotein metabolism, which prompted us to conduct this work.

As the results show, adolescents with obesity had 67% higher levels of hepcidin compared to their lean counterparts, which is in agreement with data from the literature [14,15,16]. These results could be attributed to several factors, including (a) greater chronic inflammation in adolescents with obesity as evidenced by higher levels of IL-6 [17]; (b) greater visceral adipose tissue mass, which has 4-fold more crown-like structures [18] that participate in the expression of IL-6 and in the expression of hepcidin in adipocytes [9]; and (c) greater insulin resistance [15], further aggravating the chronic inflammation, atherogenic dyslipidemia and visceral adipose tissue hypertrophy.

Along those lines, our adolescents with obesity displayed higher levels of IL-6 as also shown before by others [15,19], arguing for a plausible causative role for the observed increase in hepcidin. However, physiologically, hepcidin is the master regulator of iron stores, curtailing the entry of iron to plasma when the signals of iron excess are appropriate. In fact, higher hepcidin levels may indicate iron overload [5].

However, considering the poor diet of our population, iron overload is unlikely. As hepcidin sits at the intersection between iron status and inflammation, it became imperative to assess iron stores in our population, since hematological indicators are not sensitive enough. Moreover, it was necessary to rule out the confounding role of iron status on the metabolic findings in our study. Indeed, several authors have precisely identified hepcidin as a putative causative factor for anemia in obese adults and adolescents [2,6,7,8]. Thus, to clarify whether the higher hepcidin values we found were due to impaired iron metabolism (namely, iron overload) or, on the other hand, were already causing iron deficiency, we evaluated three key markers of iron status: ferritin, transferrin and soluble transferrin receptor, which can also provide an assessment of the distal consequences of the elevated hepcidin levels we found. In fact, ferritin levels in adolescents have been suggested by others as an important early indicator for the risk of developing metabolic disorders [20,21]. Another aspect that deserves further attention in the light of these findings is the interaction between copper and iron metabolism. Copper deficiency has been shown to aggravate the impact of fructose in NAFLD [22].

Current recommendations favor TBI as the most accurate marker of iron status [13,23]. NHANES used the TBI model, in which the log ratio of sTfR to serum ferritin is assessed. Together, sTfR and ferritin concentrations cover the full range of iron status; moreover, they better predict bone marrow iron. Besides, TBI can be analyzed as a continuous variable [13,23].

Ferritin was higher in our adolescents with obesity as compared to their lean counterparts. Ferritin is a good indicator of iron stores provided there is no inflammation, as it is also an acute-phase reactant [24]. In the light of increased IL-6 levels, ferritin levels become difficult to interpret, but are more suggestive of chronic inflammation rather than iron overload and, to a certain extent, surrogate markers of MetS [24].

No differences were found for transferrin, soluble transferrin receptor (sTFR, sTFR/ferritin, log sTFR/ferritin, or sTFR/log ferritin between our two cohorts. These are far more sensitive than standard serum iron and transferrin and have a very good correlation with the invasive gold standard that requires bone marrow aspiration with iron staining, which is obviously out of the question for a study like this. These data provide strong evidence for the absence of differences in TBI between our two populations, even on a terrain of increased ferritin in obese children, arguing for inflammation as the most plausible main cause in this case.

**Figure 2:**
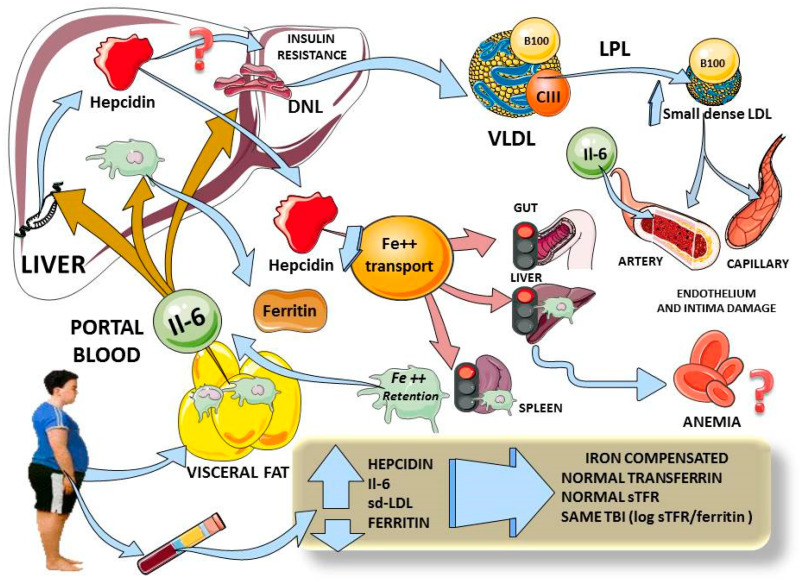
Diagram summarizing our findings and plausible physiological mechanisms. Adolescents with obesity have increased visceral fat, and we found increased levels of IL-6 which, via portal circulation, reach the liver to simultaneously induce hepcidin expression, Kupffer cells’ ferritin secretion and hepatic insulin resistance (3 beige arrows). Higher levels of hepcidin would curtail iron entry from gut, liver and spleen macrophages (pink arrows) which, in chronic stimulation, would lead to anemia. Moreover, iron retention activates macrophages to secrete even more IL-6. Hepatic insulin resistance induces lipogenesis, leading to high serum VLDL and small dense LDL, which are atherogenic and damage endothelium. In adolescents with obesity, we demonstrated increases in hepcidin, IL-6 and small dense LDL, all correlating with hepcidin. Ferritin levels were also high, but, as shown by total body iron measurement, did not appear to account for iron overload but rather for inflammation. All indexes of iron status showed no difference between lean and obese participants. This diagram was designed in part using Servier Medical Art: https://smart.servier.com (accessed on 29 November 2020). DNL: de novo lipogenesis; VLDL: very low density lipoprotein; sd-LDL: small dense LDL; TBI: total body iron; sTFR: soluble transferrin receptor.

Considering the above, and as we summarize in Figure 2, hepcidin elevation in adolescents with obesity appears to be linked to increased inflammation (IL-6), increased insulin resistance and visceral fat. This is in agreement with the data found by others in adults [6]. The changes in hepcidin do not appear to be due primarily to iron overload, since the other markers of iron metabolism were not different between the groups.

We posit that the most plausible primary mechanism in play is the interplay between portal blood IL-6 (and other cytokines), stemming from visceral fat and acting on the liver to produce IR directly and via enhanced lipogenesis. Lipogenesis and IR lead to cardiometabolic dyslipidemia. In parallel, the same increased portal IL-6 induces hepcidin expression and secretion. In our population, these increased hepcidin levels have not yet produced iron deficiency, but are prone to do so with time, as shown by others in adults and adolescents [6,7,8,9].

As limitations, we grant that we had a relatively small sample size (which reduced the range for some correlations). Serum iron and transferrin saturation could add to this study, but they require a greater sample volume than what we had in this ancillary study and, while they are clinically very useful, they lack sensitivity, and therefore would not likely change our conclusions. We did not have access to DEXA for more accurate measurement of visceral fat but substituted it with VAI, which is a good surrogate. We acknowledge that the crossroads of inflammation, IR and iron metabolism are complex networks that deserve further studies to elucidate. However, this does not detract from the novel and significant substantial differences we report in this study on hepcidin, visceral adiposity and triglyceride-rich lipoprotein dysregulation.

## 5. Conclusions

This study shows for the first time the association of hepcidin with triglyceride-rich lipoprotein dyslipoproteinemia, small dense LDL and IR in adolescents, showing major changes in adolescents with obesity.

## Figures and Tables

**Table 1:** Clinical characteristics of adolescents.

	Control Group *n* = 30	Obesity Group *n* = 29	*p*-Value
Female/Male	14/16	13/16	NS
Age (years) *	16.5 (15.0–18.0)	17.0 (16.0–18.0)	0.69
Height (cm)	162.8 ± 7.4	166.0 ± 8.5	0.131
Weight (kg)	58.1 ± 7.9	89.5 ± 14.9	<0.00001
BMI (kg/m^2^)	21.9 ± 2.0	32.3 ± 3.8	<0.00001
Waist circumference (cm)	72.9 ± 5.4	98.6 ± 9.9	<0.00001
Hip circumference (cm)	93.4 ± 6.2	112.0 ± 8.1	<0.00001
VAI	0.9 ± 0.6	1.6 ± 0.8	<0.001
Body fat %	24.2 ± 6.8	36.7 ± 7.9	<0.00001
SBP (mm/Hg)	110.1 ± 8.1	116.9 ± 7.4	0.001
DBP (mm/Hg)	68.8 ± 7.0	73.0 ± 7.1	0.029

*p*-Values were calculated using the Student’s *t*-test for variables with normality or the Mann–Whitney U test * for non-parametric variables. BMI: body mass index; VAI: visceral adiposity index; SBP: systolic blood pressure; DBP: diastolic blood pressure.

**Table 2:** Iron metabolism markers.

	Control Group *n* = 30	Obesity Group *n* = 29	*p*-Value
Hepcidin (pg/mL)	8419.1 ± 4826.8	14,070.8 ± 7113.5	<0.0007
IL-6 (pg/mL) *	0.9 (0.5–1.3)	2.0 (1.1–4.9)	<0.0001
Ferritin (pg/mL)	55.1 ± 39.6	94.4 ± 82.4	0.024
Transferrin (μg/mL)	3199.8 ± 1131.7	2983.5 ± 640.8	0.380
sTFR (μg/mL)	1.6 ± 0.6	1.8 ± 0.6	0.260
sTFR/ferritin ratio *	0.03 (0.02–0.05)	0.03 (0.01–0.05)	0.388
log10 sTFR/ferritin ratio	−1.5 ± 0.4	−1.6 ± 0.4	0.197
sTFR/log ferritin ratio	1.1 ± 0.8	1.0 ± 0.4	0.651

*p*-Values were calculated using the Student’s *t*-test for variables with normality or the Mann–Whitney U test * for non-parametric variables. sTFR: soluble transferrin receptor.

## Data Availability

Data are available upon request to the corresponding author.
